# Women’s Experiences Regarding Maternity Care in a Selected Hospital in Vhembe District, Limpopo Province: A Qualitative Approach

**DOI:** 10.3390/healthcare12232341

**Published:** 2024-11-23

**Authors:** Tshiembe Masibigiri, Azwinndini Gladys Mudau, Duppy Manyuma

**Affiliations:** Department of Public Health, Faculty of Health Sciences, University of Venda, Thohoyandou 0950, South Africa or 11629526@mvula.univen.ac.za (T.M.); azwinndini.mudau@univen.ac.za (A.G.M.)

**Keywords:** experience, maternity care, women, quality

## Abstract

Introduction: A pregnant woman’s life and that of her child largely depend on the care they receive during the critical stage of pregnancy, labour, and the postpartum period. Some women question their decisions regarding future pregnancies as well as where and from whom they will receive their pregnancy care because of negative experiences that they have had. Aim: The aim of this study was to explore and describe the experiences of women regarding maternity care services in a selected hospital in Vhembe District, Limpopo Province. Methods: A qualitative approach using a phenomenological research design was adopted in this study. The study population included pregnant women who were admitted to the maternity ward of the selected hospital. A total of 18 participants and 1 hospital were purposively sampled. Pre-testing was conducted to check if the central question was clear and to test the researcher’s interviewing skills. Face-to-face interviews were conducted with all sampled participants in April and May 2024. Interpretive analysis was used to analyze the data collected from the participants. Results: Four themes emerged during data analysis: women’s experiences regrading the provision of maternal healthcare; environmental factors affecting maternal health services; lack of resources; and the attitude of nurses towards patients. Conclusions: This study concludes that the majority of the women stated that they were not happy about the maternity care services provided at the selected hospital. Issues such as trained nurses being verbally and physically abusive towards women in labour, as well as the infrastructure and lack of resources of the hospital contributed towards women not being happy about the provision of maternity care services being provided. This study recommends that in order for maternity care services to improve at the selected hospital, there should be workshops for staff on how to improve patient care principles and improvements in the standard of cleanliness in and around the selected hospital.

## 1. Introduction

Maternity care refers to any care provided throughout pregnancy, labour, and the postpartum period. A woman’s life and that of her newborn depend on receiving adequate maternity care [1]. Services such as antenatal care are provided for by skilled healthcare workers and are crucial in detecting any complications during pregnancy, caring for any illnesses that may arise during the pregnancy, and helping to reduce the chances of maternal death; thousands of women and newborns succumb to preventable deaths due to pregnancy and childbirth-related complications [2]. To ensure the safety of the pregnant woman and that of her child, adequate care should be provided during the entire pregnancy, the labour period, and postpartum [1].

A study conducted in Australia by Hargreaves et al. [3] reported that most women felt isolated and confused by the care they received from their caregivers. There was no effective communication between the women who were giving birth and their caregivers, and those who were not giving birth for the first time stated that they did not feel prioritized and indicated that they would have preferred to have gone to a different hospital where private care might have been different from the maternity care they had been receiving in public hospitals. Women expressed having felt trapped because the midwives who provided their care would mostly talk at them without providing enough information about what procedures were being performed on them or for what purpose, as opposed to talking with them and providing details as to why certain things were necessary. Their overall experience was not pleasant.

According to a study conducted in the United States of America by Tikkanen et al. [4], most women noted that mental health issues and breastfeeding aid were not addressed, though their labour process was safe and satisfying with good maternal care. Hospital births were advocated as opposed to home births.

Redshaw et al. [5] conducted a study in England and found that the majority of the women expressed how they felt they had received adequate care during pregnancy and labour; they felt proper communication had been established with them during labour and the birth. However, once they had given birth, the majority of women indicated that healthcare professionals treated them as though they were no longer important.

Hameed et al. [6] reported that violation of rights and mistreatment were most prevalent amongst underprivileged and poorer women in Pakistan. Underprivileged women stated how they experienced discrimination and both verbal and physical abuse in public health facilities.

Respectful maternity care is a big challenge in Afghanistan. As stated by Manalai et al. [7], violation of respectful maternity care is largely due to a lack of awareness, excessive workload, and a lack of resources. Women expressed being mistreated and physically and verbally abused in healthcare facilities while giving birth.

Women in the north and upper regions of Ghana stated how they received less care, there was a shortage of skilled attendance, drugs were not readily available, and there was little accountability and insufficient efforts made to improve the quality and equity of care [8]. In a country like Tanzania, mothers and their newborns often receive poor-quality care, as the increased utilization of care services has failed to elicit the expected corresponding increase in survival [9].

The guidelines for maternity care in South Africa [10] stipulate that every healthcare provider who is administering care to a pregnant woman must show respect and genuine interest in their clients, without being rude, arrogant, dismissive, or judgmental. This quality of care must be provided regardless of a poor working environment or perceived unsafe practises engaged in by the pregnant woman. A study conducted by Ramavhoya et al. [11] indicates that the guidelines for maternity care in South Africa are mainly administered by midwives who serve as the primary providers of maternity healthcare services. Although these primary healthcare providers are offered training on how to meet the guidelines for maternity care, such services are still poorly implemented. Issues such as lengthy waiting times for patients, a lack of human resources, and reduced involvement of the community influence the quality of care being provided, which should be in accordance with the guidelines for maternity care.

Maternal care is essential in ensuring the health and well-being of a pregnant woman’s life and that of her child. Many studies have assessed how maternal healthcare services can be implemented to ensure quality care for pregnant women [11]. This study sought to explore and describe the experiences of women regarding maternity care services in a selected hospital in Vhembe District, Limpopo Province.

### Definition of Concepts

- Experiences

The conscious contact of facts or events that can be lived or observed, Hesse [12]. In this study, experience refers to how a woman has lived through any maternity care service in the selected hospital in Vhembe District, Limpopo province

- Women

An adult female, Collins [13]. Meyer [14] refers to women as having a biological, anatomical, and genetic identity. In this study, a woman refers to any pregnant female who has just given birth in the selected hospital in Vhembe District, Limpopo Province.

- Maternity care services

Maternity care services refer to any care provided during pregnancy, labor, and the postpartum period. As stated by the guidelines of maternity care in South Africa [10], maternity care is provided to all pregnant women in South Africa and is a free service that is there to enhance the basic needs of primary health of pregnant women. In this study, maternity care services will mean any form of service rendered by healthcare workers to pregnant women during labor and the postpartum period.

- Healthcare workers

A healthcare worker works jointly, interrelatedly and, flexibly with patients with the common goal of assisting them with assessment, treatment, intervention, and rehabilitation, Babiker et al. [15]. In this study, healthcare workers refer to a nurse (registered or enrolled) and doctors who are working in the maternity ward.

## 2. Materials and Methods

A qualitative approach and a phenomenology design were used as described by Brink et al. [16] and Maree [17]. A qualitative approach was used to explore and describe in-depth information about women’s experiences regarding the maternity care services they received before, during, and after childbirth. A qualitative approach allowed the researcher to gather information from the participants without any limitations, and a phenomenology research design was used [18].

A phenomenological design was used as it provides an opportunity to study the human lived experiences in certain environments [16]. Making use of this design was the best approach to explore and describe the experiences of women regarding maternity care services they had received in a selected hospital in Vhembe District, Limpopo Province.

### 2.1. Study Setting

This study was conducted in Vhembe District, Limpopo Province, at a selected hospital. Vhembe District is situated in northern Limpopo and is predominantly a rural area. There is 1 regional hospital, 6 district hospitals, 8 community health centers, and 1 specialized psychiatric hospital [19]. This study was conducted at a regional hospital, which is a second-level healthcare that receives referrals from and provides specialist support to district hospitals. The selected hospital offers clinical services such as surgery, obstetrics and gynecology, outpatients departments, medicine, pediatrics, mental health, geriatrics, casualty, and clinical forensic medical services [20].

### 2.2. Recruitment and Sampling of Participants

Permission to conduct this study at the selected hospital was obtained from the regional and district levels of the Department of Health. Arrangements were made with hospital managers that the researcher would be there during the week for as long as needed to collect data from the maternity ward. The researcher visited the maternity ward and approached women who had given birth and were being discharged. The researcher was the main instrument for collecting data. The researcher informed the potential participants that participation in this study was voluntary, and it could be terminated at any time should they wish to do so, anonymity would be maintained in that no one’s names would be used, the interviews would be private, the interview would be recorded on a tape recorder and freehand notes would be transcribed strictly to retain useful information. Participants were sampled purposively [21].

### 2.3. Ethical Considerations

Ethical clearance was obtained from the University of Venda ethics committee (FHS/23/PH/26/0602), Limpopo Department of Health, Vhembe District Department of Health, and the selected hospital issued the researcher with letters to allow the researcher to recruit participants from their institutions. All participants were issued with consent forms and information sheets regarding this study. The researcher ensured that no participant was physically, psychologically, or emotionally harmed during participation in this study. The participants’ names were not included in this study.

### 2.4. Data Collection

Participants were approached, and individual face-to-face interviews were conducted by the researcher in a private cubicle in the postnatal ward at the selected hospital in April and May 2024. An interview guide [16] was used to conduct interviews with participants; the interview guide consisted of the participants’ demographic information, such as their age, mode of delivery, and how many times they have been admitted to the maternity ward of the selected hospital. One central question was asked, “May you please share your experience regarding the maternity care you have received since you arrived at the hospital until now?”. This was followed by probing questions depending on what the participants’ responses were. Field notes were taken, and the interviews were recorded. Skills such as probing, paraphrasing and summarizing were used in order to understand the experiences of women regarding the maternity care services that they have received. Data were collected in English and Tshivenda, and the data collected in Tshivenda for participants who could not speak English were translated into English and then transcribed verbatim. Interviews were conducted until no new information was found. Data saturation was reached by Participant fourteen, but the researcher continued with research interviews until Participant eighteen to ensure that no new information was found. Each interview lasted approximately 30–45 min.

### 2.5. Data Analysis

The interpretative phenomenological approach by Engward et al. [22] was used to analyze data from women through their individual interviews. The researcher read and reread through all the transcribed data to gain a better understanding of the participants’ responses. The transcribed data were then arranged into categories and subcategories and labeled according to the participants’ responses during the interview. Once categories and subcategories had been arranged, the researcher then came up with themes which were the major findings of this study. Four themes were generated in this study: women’s experiences in the provision of maternal healthcare, environmental factors affecting the provision of maternal healthcare services, and lack of resources and attitudes of nurses towards patients. The researcher interpreted the findings of this study based on women’s experiences regarding maternity care services in Vhembe District, Limpopo Province. The researchers’ interpretations are based on women’s experiences regarding the maternity care services they have received.

## 3. Results

The demographic information of the 18 participants who were interviewed in this study is presented in Table 1 below. The table lists the participants’ age, mode of delivery, and the number of times they have been admitted to the selected hospital’s maternity ward.

From Table 1 above, the demographic information revealed that of the *n* = 18 participants who were interviewed in this study, most, 72.22% (*n* = 13), gave birth through natural vaginal birth, while a minority, 27.78% (*n* = 5), gave birth through caesarian section. Most of the participants were of Venda ethnicity 72.22% (*n* = 13), followed by Tsonga ethnicity 22.22% (*n* = 4) and Shona 5.56% (*n* = 1). Participants ranged from 19 to 38 years, with a mean age of m = 28.72 and a standard deviation (Std) of 6.43. The participants differed in the number of times they were admitted to the hospital, ranging from 1 to 4, with a mean admission of 2.11 and a Std of 1.16.

Four main themes emerged from qualitative data collected through interviews with the participants: women’s experiences regarding the provision of maternal healthcare, environmental factors affecting maternal health, lack of resources, and attitudes of nurses towards patients.

Theme 1: Women’s experiences in provision of maternal healthcare

The standard of care that participants experienced was not the same. Almost all the participants indicated that the care they received after childbirth was better than the care they received during the delivery process. Most of the participants explained that nurses checked to see how many centimeters along their dilation was, their blood pressure and the babies’ heartbeat regardless of whether the nurse was friendly or rude.

Prenatal care

With regards to having vitals checked and tests being performed regularly, nurses seem to be able to attend to their patients.

**Participant R**: *“…I was admitted because I had to have a C-section because this was my second baby. I only ended up getting the C-section done 3 days later because there were other emergencies ahead of me. The doctor explained to me how my water hadn’t broken yet and that the reason for the delay was that other patients had to be rushed to the theatre due to life threatening emergencies. The nurses checked my BP, urine, and blood daily and were very friendly towards me. I had such a nice stay here; everyone made me feel comfortable….”*

**Participant A**: *“…The nurses checked my BP and told me to come to her every 2 h so she can check for the baby’s heartbeat. And that if I start feeling a lot of pain or if I think I want to go to the toilet I should call her she will come…”*

Not every participant had the same positive feedback with regards to being assessed by nurses.

**Participant I**: *“…When I arrived, no one attended to me or checked me for over 2 h. When a nurse eventually came, I told her I felt like I needed to go to the toilet, and she told me to lie down its not time. I didn’t know what to do, it was an older nurse, so I was too scared to answer back or say anything…”*

**Care during active labour**

The findings of this study revealed that issues between the patients and the nurses arise when the pregnant mother must deliver the baby. Most of the participants shared how the delivery process was very hard and even more unbearable because of how nurses treated and responded to them at that time. It seems that nurses are very rude and do not seem helpful or supportive to patients when it is time for them to deliver their babies.

**Participant E**: *“…When it was time for the baby to come the nurse told me to get on the bed, I was in a lot of pain, and I had forgotten I had my phone in my hand. The nurse then said I must get off the bed and go put my phone in the drawer because I want to record her. I asked the nurse to put it away for me and she refused and insisted that I get off the bed and do it myself, which I refused because I could feel the baby’s head was coming out and I didn’t want my baby to fall on the floor. The nurse said she wouldn’t help me because I am being cheeky so I started shouting at her saying she must help me, and I threw my phone at her legs. Only then did she assist in delivering my baby…”*

**Participant O**: *“…The nurse was very rude, she accused me of wetting myself when my water broke. Yooh! It was so bad. That nurse didn’t even tell me to get on the bed and she could see I was in a lot of pain, she just kept shouting at me, I just wanted to cry. When the baby came out, she said to me look its boy I’m sure you will keep coming back here because you are looking for a girl. It was so painful. I felt so down and lonely…”*

The poor service provided by nurses during labour is also shown in the following statement.

**Participant I**: *“…When it was time to push, I was so scared, the nurse kept shouting at me so loudly I don’t even know what she was saying because I was so scared and confused and just did not know what to do. After the baby came out, I had to get stitches and I was crying because it was so painful, but the nurse kept yelling for me to keep quiet I’m making noise…”*

**Postnatal care**

Participants shared how they felt that nurses are mainly nice and friendly only after they have given birth. None of the participants expressed any bad behaviour or ill treatment once in the postnatal ward. Participants seem to have felt well taken care of, and nurses showed interest and concern about their well-being and that of their baby.

**Participant P**: *“…I am honestly relieved to have given birth. The nurse checks on me and the baby. She also showed me how to hold the baby so that she can breastfeed well. It’s surprising how nice the nurses are this side…”*

Nurses were not only helpful but also gave helpful advice to participants about how to care for their baby.

**Participant L**: *“…I had dozed off and the baby woke up, that nurse told me to wash my hands first so I can pick up the baby. She advised me that I must wash my hands often before picking up the baby so that she does not get germs or get sick. She even showed me how to hold the baby’s head properly see just look…”*

Theme 2: Environmental Factors affecting Maternal Health Services

This study revealed what participants thought about the overall environment of the hospital and many of them expressed their disappointment about the hospital. They said that the building itself looks very old and bushy, they mentioned stains in the bathrooms, and described their stay at the hospital as like living in the bushes. They also mention the presence of animals that roam through the wards, and how this makes them question how clean the hospital is when providing care for sick people.

Hygiene

The hospital does not appear to be clean to most of the participants, and they expressed how little effort is made to maintain a clean environment where patients can live once they have been admitted into the wards. There is a huge problem with regard to the level of hygiene being maintained. A participant expressed how the ward looked very dirty and that she had not seen anyone come and sweep or mop the place since she was admitted; she was extremely unhappy about the place being dirty.

**Participant N**: *“…Since I arrived yesterday until now, I have not seen anyone cleaning here. This place is very dirty…”*

There were stains in the bathtubs and in the toilets; it was very uncomfortable for patients to use these facilities, and they felt that it was too unhygienic a place for newborn babies.

**Participant M**: *“…It’s not clean here at all, quite dirty. Even the stains in the bathrooms are so bad I don’t even want to sit on the toilet. This is not a clean place for a baby to be in…”*

Participants expect a certain level of cleanliness in a hospital, and mention how current cleaning practices are very confusing and unsettling for patients when they see no difference.

**Participant A**: *“…I don’t understand why this place looks so untidy, I have seen cleaners come in and mop, but it still looks dirty…”*

Animals in the ward

Every participant spoke about how monkeys and cats roam freely within the ward at any time of day. Staff make no effort to chase them away. And if a patient is not careful and leaves food unattended, monkeys have been seen to take patients’ food, or you cats eat patients’ food.

One participant explained how she had left her food unattended for less than 5 min and when she came back, her plate had been taken by a monkey.

**Participant B**: *“…There are cats and monkeys that get in the ward, and no one is chasing them away. I left my food on this table, and I went to the toilet, when I came back my food was gone. A lady next to my bed showed me a monkey outside that was eating and said it came in and took my food and it’s eating it…”*

The animals in the ward seem to be a problem for patients, especially those who do not like cats. One participant felt that it was not safe to have animals in the ward or to expect patients to sleep on beds where cats or monkeys had been sleeping or sitting without linen being changed.

**Participant F**: *“…I don’t like cats at all and there are so many here. You will find a cat sleeping on the bed and the nurses will say go sleep on it without even changing the linen. It’s very bad…”*

**Participant J**: *“…There are a lot of cats everywhere…”*

Another participant said that she was disgusted by seeing cats eating food that had been prepared for patients within the ward; and for those reasons, she refrained from eating any food served by the hospital and relied on food brought from home.

**Participant O**: *“…There was food in that tray which had been left by the entrance and a cat was busy licking that food. When the lady came back, she just hit the cat with a book and it ran away, but she proceeded to hand out the same food to patients in the ward. That is why I have not been eating the food here…”*

Infrastructure and surrounding

Participants were not impressed with the overall environment of the hospital. The hospital was described as old and falling apart; it was not well maintained. A participant mentioned that parts of the ceiling in the ward had come off and that the structure of the entire hospital building looked too old in such a way that it might collapse with people inside.

**Participant J**: *“…This building looks like it can fall any time, just look at that ceiling coming off. This place is old…”*

There are huge trees and thick high bushes that surround the hospital, and within the hospital grounds, the grass is very high. The area is very dense and bushy, and this has a participant worried about safety with regard to animals such as snakes being a common thing in such a bushy environment.

**Participant H**: *“…This hospital is too bushy, it’s almost as if it’s a forest. What if there are snakes here and come into the ward and bite us…”*

Theme 3: Lack of resources

Patients mention that the hospital seems to be lacking some resources, such as pads and blankets, and that there have even been water shortages. Interviews also revealed that patients had to take turns to bathe because water sometimes runs out, and so patients have to wait until there is water to bathe. This also affects the functionality flushing toilets.

Materials

Participants shared how they were not given enough pads after giving birth, how there was one thin blanket on each bed, and that the ward was very cold. It was also stated that newborn babies were not given warm blankets to be wrapped in.

Participants expected to have a bed with a warm blanket for them to sleep in, but the hospital only provides one thin blanket.

**Participant J**: *“…There are no blankets in this hospital, they gave us thin ones and it gets very cold at night…”*

**Participant A**: *“…These beds don’t have proper blankets it gets very cold at night…”*

Some participants expected the hospital to offer them pads after they had given birth. They were only offered pads after the delivery of their baby, but when more were needed, the participants were told that there weren’t enough pads anymore, so they couldn’t be given any more.

**Participant F**: *“…After I gave birth they gave me a pad, but I was bleeding a lot, so I asked the nurse for another pad, and she told me there aren’t enough pads to be given more. Just imagine Yooh…!”*

Shortage of water

The hospital does not always have running water. There are times when water has run out or the supply is cut off, so patients cannot bathe or flush the toilets. This becomes a huge health concern and culminates in a dirty and smelly ward.

**Participant D**: *“…There is only cold water to bath in. in the morning I had just gotten in the shower then the water was finished before I could finish bathing. When I told the nurse about the water not coming out, she said it’s just finished I must wait maybe an hour then I can go bath to bath. The toilets weren’t flushing either, so it was so smelly and dirty until the water came back…”*

A lack of water compromises the level of care and hygiene within the hospital as patients are unable to keep themselves clean or after giving birth and the ward begins to smell when they cannot flush the toilets. Patients should be able to have access to clean water when they have been admitted in the ward instead of having to ration how and when water can be used.

**Participant B**: *“…The nurse told us that we had to wait and take turns to bath because water won’t be enough for everyone, and it gets finished…”*

Theme 4: Attitudes of nurses towards patients

This study revealed that patients are not receiving the best level of care in the maternity ward. Participants shared how they were verbally and physically abused by nurses. The nurses that attended them were portrayed as having bad attitudes. Preferential treatment was given to Indians while Zimbabweans were ill treated. Information regarding the health status of a patient is not always communicated or shared with patients, though some patients were well informed about what was happening and why it had to be that way.

Physical and verbal abuse

The findings revealed that the majority of the participants had poor experiences due to the bad attitudes of the nurses; some reported being too terrified to ask for help or assistance due to the attitude and behavior of the nurses towards them and other patients who were in the ward. Participants said nurses were rude, abusive, and mistreated them.

**Participant C**: *“…I was feeling so much pain and could feel the baby was coming, then I called out to the nurse to come and help me because the baby was nearby. The nurse then started shouting at me saying I am making noise how do I know the baby is here because they have already checked me. I just didn’t know what else to do because the pain was just too much painful…”*

The nurses displayed verbal and physical abusive towards their patients.

**Participant F**: *“…I was screaming because of the pain. The nurse came and checked me and told me the baby was still far. The pain just kept coming and I was calling out to the nurse, when she came back, she was slapping my back very hard saying I should stop making noise she wasn’t there when I made the baby and she left. I was walking around in the ward making a lot of noise because of the pain then I got on the bed because I could not walk anymore, then I called for the nurse, and no one came. I started to feel this big pressure and pain and I was screaming even more loudly, and the baby came out right there on the bed. I was so scared because I was alone. The nurses ran to me and were shouting at me saying what did I do? Who told me to push the baby? They were very angry at me…”*

The abusive behaviour that nurses displayed is very intimidating towards other patients and makes it very difficult for patients to willingly go seek help from the nurses even though they are in pain and do need medical assistance.

**Participant K**: *“…After I got my file, I arrived at that ANC ward and I found a nurse shouting at this other lady, I got so scared, so I just sat on the bench by the entrance. Maybe an hour went by, and no one came to me, and I knew the nurses saw me, so I went over to them. The nurses asked me why I just came and sat by the door without saying anything. I hesitated and said I was scared because you were shouting at that girl over there. They just laughed and said “is this how I behave at home? Just enter a house without greeting naa?” I gave them my file and one of the nurses showed me to a bed and she checked me. They were nice to me shem. Maybe it’s because I was following the instructions they were giving. They didn’t give me any attitude at all…”*

However, one participant said that she was treated well by a nurse.

**Participant M**: *“…The nurse helped to get on the bed because I was not feeling well, my stomach was very painful, but she told me that I should try lie down since I’m only 2 cm and to call for her if the pains get worse. She was so nice to me…”*

Unfair discrimination and ill treatment

A few participants indicated that nurses treated people differently based on whether they are Zimbabwean or Indian. It seems that patients felt that they were not all treated equally and this very unfair.

**Participant G**: *“…The nurses must treat us all the same, there’s no need to make Indians feel better than us. We all the same here…”*

The unfair treatment seemed to be based on discrimination; one Indian patient in the ward seemed to have been given special treatment over everyone else.

**Participant H**: *“…These nurses are somehow. They tell us visiting hours is only at 12 but that Indian over there had visitors come in when it was 3 and they were many people almost 10 busy crowding the ward and making noise, but the nurses didn’t say anything. When we get visitors, they will say we can only allow 2 people inside then they tell them to leave at 1. It’s not nice when they do these sorts of things…”*

A participant alluded to the fact that she was treated poorly because she is Zimbabwean.

**Participant C**: *“…When my water broke, I was standing by a bed, and it made a big mess. The nurse was so angry busy shouting at me saying you Zimbabweans like coming here to give birth, look at the mess you have made tomorrow you want to go on radio saying we are bad but here you are busy making all these mess. You people must go home and start giving birth there. I felt like crying. Another nurse came and checked me then she said it doesn’t look like there is a way for the baby I will have to go for C-section, this was so scary for me…”*

Lack of transparency

Interviews revealed that participants all wished to know what was happening when the doctors and nurses attended them. They prefer to have the full details concerning their health and the health of their baby. Some participants were never told why certain procedures were being performed or the purpose of such procedures.

**Participant J**: *“…The nurse was rude. I didn’t even know how many centimeters I was every time she would check me. When I asked her how long she thought it would take for the baby to come she didn’t answer. Even when she was taking my blood, she didn’t say what the bloods tests were for. Yoooh! Ha ah, these people are somehow…”*

Conversely, another participant expressed how she had felt relieved during her C-section when doctors paid attention to what she was saying and made sure she was well medicated throughout the procedure.

**Participant Q**: *“…I was admitted because it was an emergency and the doctor told me I was going to get a C-section because the baby’s heartbeat was very low, but not to worry too much. When I was inside the theatre, they started to cut me and I told the doctor that I can feel what they are doing, he then told me that they are going to increase the dose so that I won’t feel anything. After a little while he asked me if I could still feel anything, and I said no. Shooo! It was such a relief. Afterwards when I woke up, they brought my baby and told me that the operation was a success. I was very happy…”*

## 4. Discussion

The findings of this study revealed that most of the women received good-quality prenatal care. Continuous assessment of vitals was performed by nurses. According to Cardona-Moreell et al. [23], the continuous assessment of vital signs in patients by nurses guarantees that the patients will have a more clear and detailed medical history and early detection of potential health risks.

Good prenatal care assists nurses in being able to foresee any possible complications that may arise with their patients, and it also affords their patients peace of mind that their health needs are being met. Liu et al. [24] illustrated that good prenatal care not only benefits the infant but the mother as well, especially women who had vaginal deliveries. Having experienced good prenatal care also reduces the chances of postpartum maternal hospitalization. This creates a positive impact on infant and maternal health.

The major problem seems to arise during the active labour stage; all participants expressed how nurses were rude, and abusive, used derogatory language, and made their childbirth process very stressful and unbearable. Zitha and Mokgatle [25] show how midwives’ attitudes and behaviour were abusive and left women fearful and anxious; this also made some women push the baby before it was time to receive attention and care from the midwives. The childbirth process felt very strenuous and hostile to the pregnant women.

During the postnatal stage, this study revealed how women had a positive experience and that nurses were helpful and kind towards them and their infants. There was continuous assessment of both infants and the moms; and women felt that their needs were being met.

Environmental Factors Affecting Maternal Health Services

This study has revealed that for patients to receive good-quality maternal care, the environment of the hospital and its surroundings play an important role in providing quality care. The selected hospital looks dirty; patients reported dirty bathrooms that are full of stains. Another patient reported that she had not seen any cleaners come to clean the ward she was in since she was admitted. The hygiene level is very poor and unsanitary, especially for a ward where there are newborn babies. There is a high chance of bacteria and/or infection spreading due to the uncleanliness of the wards. Bouzid et al. [26] state that one of the main cornerstones of a safe environment is cleanliness and the maintenance of hygiene. Health facilities need to be able to offer their patients a clean hygienic environment to reduce the spread of viruses and to guarantee the health and safety of their patients.

This study showed how animals were a big problem at the hospital where this study was conducted. Monkeys and cats roam freely and unsupervised through the wards. No one restricts or chases these animals away. Cats and monkeys sleep on patient beds, which is very unsanitary, and linen is not changed afterwards. Food is left unattended, and cats and monkeys eat or lick this food that is then still offered to patients to eat. This is unhygienic and not safe. Pathogens can be transmitted from cats or monkeys to patients, who might then become unwell. The presence of these animals in the ward greatly increases the risk of diseases spreading. Murthy et al. [27] conducted research stating how animals may be present in healthcare facilities for various reasons; however, to reduce the cross-transmission of pathogens from animals to human beings, there must be little to no animals within health facilities. This is to ensure the safety and well-being of individuals within health facilities.

Another environmental issue that was revealed by this study is the poor infrastructure. The hospital building structure is not well maintained. The hospital looks old and worn out; parts of the ceiling are falling off in the ward, and the hospital ground is bushy with grass at high levels. Patients expressed how the area does not feel safe with the possibility of snakes being on the hospital premises. Bouzid et al. [26] state how women in developing countries expressed how good-quality maternal healthcare service also includes a good physical environment within the health facility. This contributes to a positive experience.

Lack of resources

For a health facility to be able to offer good-quality services, resources need to be available for patients to use. Muema [28] shows that there is a relationship between the resources available and good-quality service in a hospital. For a hospital and hospital staff to deliver good maternal services, there must be availability of staff and resources to care for patients. Patients expressed how only one thin blanket was offered to them despite it being very cold at night. Patients were not warm enough. Further, essential items such as pads were lacking, and patients could not be offered more than one pad after giving birth.

A serious issue was the shortage of water at the selected hospital. Patients had to take turns to bathe due to water shortages; sometimes patients had to wait for water to be available before they could bathe. This also made toilets smelly and unclean because patients could not flush the toilets. Bouzid et al. [26] showed how women in Bangladesh, India, Thailand, and Gambia rated maternal care as good quality due to services such as the availability of water, clean drinking water and clean toilets.

Attitudes of nurses towards patients

This study revealed how nurses provide poor and low standards of maternal care for patients during active labor. The World Health Organization [2] has set out guidelines that ensure the health and safety of pregnant women and their newborns, and healthcare providers must follow these to provide a positive and supportive environment for pregnant women.

Women expressed how the nurses were not friendly or kind towards them. Patients reported being shouted at while in labour and described nurses as rude and abusive, using offensive and derogatory language towards their patients. In a study conducted by Heys et al. [29], women reported that healthcare providers who offered them care during their childbirth expressed judgment towards them, and that these healthcare providers were rude and arrogant, making inappropriate and racially abusive comments towards them. Zitha and Mokgatle [25] found that some women experienced midwives shouting at them, and rude remarks were made towards them; these left the pregnant women feeling fearful, anxious, and very nervous to be in the hospital. The hostile environment made the pregnant women feel unsafe and very scared of the midwives.

Participants gave the impression that a Zimbabwean patient was not treated well, and an Indian patient received special treatment. Visiting hours were not shared at equal allocated times. An Indian lady could have almost 10 visitors at the same time, and their visitors would stay beyond the allocated visiting time. There was unfair discrimination being practiced amongst the patients; nurses were not treating everyone the same way due to ethnicity. This discrimination could be based on nurses viewing Zimbabwean nationals as having a lower social standard than Indians. Higginbottom et al. [30] conducted a study in the UK about immigrant women’s experiences of their access to maternal healthcare. Women reported having had a negative experience in that they found health professionals to be rude, discriminatory, and very insensitive towards their cultural and social needs. This led to women losing interest in utilizing the maternity care services being provided. Small et al. [31] also conducted a study that supports how immigrant women have negative experiences due to poor communication with healthcare providers; the immigrant women perceived their caregivers to be unkind, discriminatory, and not respectful.

Lastly, this study also revealed how there is no transparency with regard to sharing information by nurses. Nurses conducted physical examinations such as drawing blood or checking how many centimeters a patient was dilated without disclosing the procedure performed or seeking consent from patients. These experiences left patients feeling very nervous and anxious because they did were not informed as to the status of their health. Jenkins et al. [32] state that for healthcare providers to ensure a positive experience for pregnant women, there needs to be open communication between the caregiver and their patients. Women seem calmer and happier when they are continually informed about the care and procedures that they receive from their healthcare providers.

## 5. Conclusions

Maternity care services are the cornerstone of ensuring the health and safety of pregnant women and their newborns. It is the responsibility of healthcare facilities and all healthcare providers to ensure that the maternity services provided to pregnant women are of good quality.

The provision of good-quality maternity care services improves women’s prospects of future pregnancies. Good-quality maternity care services also provide women with a sense of security and safety during childbirth. This also reduces childbirth complications and injuries as well as postpartum health complications.

Urbanova [33] states how the negative birth experiences of women have been shown to lead to the risk of women being diagnosed with postpartum depression. These unsatisfactory childbirth experiences leave women battling with mental health issues such as anxiety and depression.

This study has shown that healthcare providers at the selected hospital provide poor maternity care services to pregnant women during the childbirth process. There is still a gap between the care that is provided and the guidelines for maternity care that have been set out. Good-quality maternity care services not only depend on the services provided by healthcare providers, but also on a clean and safe environment.

This study was conducted at a selected hospital, so the results cannot be generalized to all hospitals in the Limpopo province.

Further, some participants refused to be recorded despite the researcher having informed them of anonymity and confidentiality, so the results cannot be generalized to all hospitals in the Vhembe District.

In conclusion, this study recommends that the Department of Health use the study findings to assess if the care provided is in line with the principles of maternal care provided and stipulated in the guidelines for maternity care. This will allow the Department of Health to bridge the gap provided and to provide good-quality maternity care services. Future researchers may also seek to enquire how good-quality maternity services are important in ensuring good physical and mental health for women who have given birth.

## Figures and Tables

**Table 1:** Demographic information of participants.

Participant	Age in Years	Mode of Delivery	Number of Times Being Admitted at the Selected Hospital	Ethnicity	Professional Who Attended the Birth
Participant A	27	Natural vaginal birth (NVD)	1	Venda	Midwife
Participant B	34	NVD	3	Venda	Nurse
Participant C	27	Caesarian section (C-section)	4	Venda	General practitioner (GP)
Participant D	38	NVD	4	Venda	Nurse
Participant E	36	NVD	4	Tsonga	Nurse
Participant F	24	NVD	1	Venda	Midwife
Participant G	36	C-section	2	Venda	GP
Participant H	32	C-section	2	Tsonga	GP
Participant I	25	NVD	2	Venda	Midwife
Participant J	22	NVD	1	Venda	Midwife
Participant K	21	NVD	1	Tsonga	Midwife
Participant L	21	NVD	1	Venda	Midwife
Participant M	29	NVD	2	Shona	Nurse
Participant N	19	NVD	1	Venda	Nurse
Participant O	37	NVD	3	Tsonga	Midwife
Participant P	36	NVD	2	Venda	Midwife
Participant Q	31	C-section	2	Venda	GP
Participant R	22	C-section	2	Venda	GP

## Data Availability

The anonymized data are available from the corresponding author upon request.
